# Molecular Mechanisms of Podocyte Development Revealed by Zebrafish Kidney Research

**DOI:** 10.4172/2168-9296.1000138

**Published:** 2014-06-07

**Authors:** R Miceli, PT Kroeger, RA Wingert

**Affiliations:** 1 Department of Biological Sciences and Center for Zebrafish Research, University of Notre Dame, Notre Dame, IN 46556, USA; 2 Department of Biological Sciences, University of Notre, Dame, 100 Galvin Life Sciences, Notre Dame, USA

**Keywords:** Kidney, Podocyte, Glomerulus, Nephron, Pronephros, Zebrafish, Retinoic acid, *Wt1a*, *foxc1a*, *rbpj*, Wt1, FoxC2, Notch

## Abstract

Elucidating the gene regulatory networks that control kidney development can provide information about the origins of renal birth defects and kidney disease, as well as insights relevant to the design of clinical interventions for these conditions. The kidney is composed of functional units termed nephrons. Renal malfunction often arises from damage to cells known as podocytes, which are highly specialized epithelial cells that comprise the blood filter, or glomerulus, located on each nephron. Podocytes interact with the vasculature to create an elaborate sieve that collects circulatory fluid, and this filtrate enters the nephron where it is modified to produce urine and balance water homeostasis. Podocytes are an essential cellular component of the glomerular filtration barrier, helping to protect nephrons from the entry of large proteins and circulatory cells. Podocyte loss has catastrophic consequences for renal function and overall health, as podocyte destruction leads to nephron damage and pathological conditions like chronic kidney disease. Despite their importance, there is still a rather limited understanding about the molecular pathways that control podocyte formation. In recent years, however, studies of podocyte development using the zebrafish embryonic kidney, or pronephros, have been an expanding area of nephrology research. Zebrafish form an anatomically simple pronephros comprised of two nephrons that share a single blood filter, and podocyte progenitors can be easily visualized throughout the process of glomerular development. The zebrafish is an especially useful system for studying the mechanisms that are essential for formation of nephron cell types like podocytes due to the high genetic conservation between vertebrate species, including humans. In this review, we discuss how research using the zebrafish has provided new insights into the molecular regulation of the podocyte lineage during kidney ontogeny, complementing contemporary research in other animal models.

## Introduction

### Kidney structure and function

The kidneys are important organs with a set of physiological roles that are essential for life [1]. Kidneys are made up of functional units called nephrons that are epithelial tubes responsible for producing urine and balancing water and salt levels [1]. One central task of the renal system is to interact with the vascular system to collect plasma from the bloodstream in order to excrete metabolic waste from the body [1]. To accomplish this task, nephrons are comprised of unique working parts located along their length. Typically, nephrons have a blood filter on one end, a duct at the opposite end that drains the urine, and an intervening segmented tubule in which domains of different cell types progressively modify the filtrate and thereby regulate solute reabsorption and secretion [1].

During development, vertebrates form up to three kidney structures of increasing complexity: the pronephros, the mesonephros, and metanephros [2]. In higher vertebrate species like mammals, the pronephros is a vestigial structure that exists transiently in the developing embryo, while the subsequent kidney forms become functional during juvenile and/or adult stages [2,3]. Other vertebrates like fish and amphibian species form only two kidney structures, and the pronephros is functional until a mesonephros is formed in juvenile stages [2,3]. Common among all of these vertebrate kidney iterations is a structural composition based on nephrons [2,3]. In addition, each subsequent renal structure that forms within any given species is more complex due to the increasing number of nephrons they contain, as well as the overall anatomical organization of nephrons. For example, early kidney structures are often comprised of parallel rows of nephrons that drain into a common duct, while later organ variations typically have branched or otherwise arborized arrays of nephrons [3].

Progress in understanding renal cell type development can be used to gain valuable medical insights for the nephrology field. Applications of such knowledge include the design of novel therapeutic approaches for kidney birth defects, as well as new interventions to treat or prevent acute and chronic kidney diseases. Defects that arise in the blood-filtering unit of the nephron, known as the glomerulus, are a prevalent initiating event common to many renal conditions [4–6]. Proper glomerulus development and maintenance of glomerular structural-functional integrity are crucial for nephron health and overall organ fitness. The glomerulus is the site where fluid is collected from the circulation using an intricate molecular sieve [7]. If this sieve is compromised, large proteins/protein complexes and circulating cells leak into the nephron tubule, a condition termed proteinuria. Proteinuria can cause damage that culminates in attrition of the entire nephron, and can initiate a vicious chronic cycle of inflammation and renal fibrosis that spreads within the kidney [4–6]. Thus, as the matrix of components at the glomerulus selectively gathers blood plasma, it serves a vital barrier function to protect the downstream components of the nephron and stave off chronic injury.

### The glomerular blood filter apparatus and roles of podocyte cells

The glomerulus is made up of unique cellular and extracellular matrix materials [7]. The renal epithelial cells located at this apparatus are called podocytes. Podocytes intimately surround the capillary tuft, forming a cellular fence that restricts fluid egress from the circulation into the nephron. Podocytes extend foot processes from their basal surface that interdigitate with the foot processes of adjacent podocytes, forming unique cell-to-cell junctions called the slit diaphragm. The interlocking network of podocytes, with these slit diaphragm junctions, are an important physical restriction on the entry of large macromolecules and hematopoietic cells into the nephron. Between the epithelial podocytes and the fenestrated endothelial cells that comprise the capillary loops is a thick Glomerular Basement Membrane (GBM) that is assembled from secreted protein contributions of each flanking cell type [8]. Together, these sandwiched layers of cells and the intervening basement membrane form an elaborate selective barrier to the entering filtrate. Genetic defects that lead to structural disruptions in any of these components can alter the integrity of the entire structure, causing proteinuria that leads to kidney disease [4–6]. There has been tremendous progress in identifying how defects in particular proteins disrupt the structure of the blood filter (both in extracellular (GBM) and cellular compartments) and trigger pathological conditions [4–6]. Nevertheless, many aspects of glomerular cell biology remain poorly understood [7], such as the identity of the genes that specify the podocyte lineage during nephrogenesis.

### Animal models of podocyte development

To date, insights into the genetic pathways that regulate podocyte development have been made through research using kidney structures in various vertebrate animal models, such as the mouse [9], due to the broad conservation of genes that exists between these species and humans. The zebrafish, Danio rerio, is a small, tropical freshwater fish that has been widely used in research to study aquatic pollutants [10] and has come to the forefront of current biomedical research [11,12] due to their substantial conservation with humans and other vertebrate species [13]. Kidney research using the zebrafish has steadily expanded in recent years due to the features of this model that enable complementary research pursuits to work in other vertebrate model systems [14–17]. The zebrafish embryo utilizes a functional pronephros, which is an anatomically simple structure comprised of two nephrons that share a common glomerulus [14–16]. The nephrons develop quickly during embryogenesis: they form and begin blood filtration during the first two days of life, thus enabling rapid interrogation of gene function tests by loss and gain of function approaches [14–16]. Further, zebrafish nephrons have a segmental organization and contain numerous cell types that are quite similar to mammals, including podocytes as well as proximal and distal tubule segment epithelial cells [17,18].

**Figure 1:**
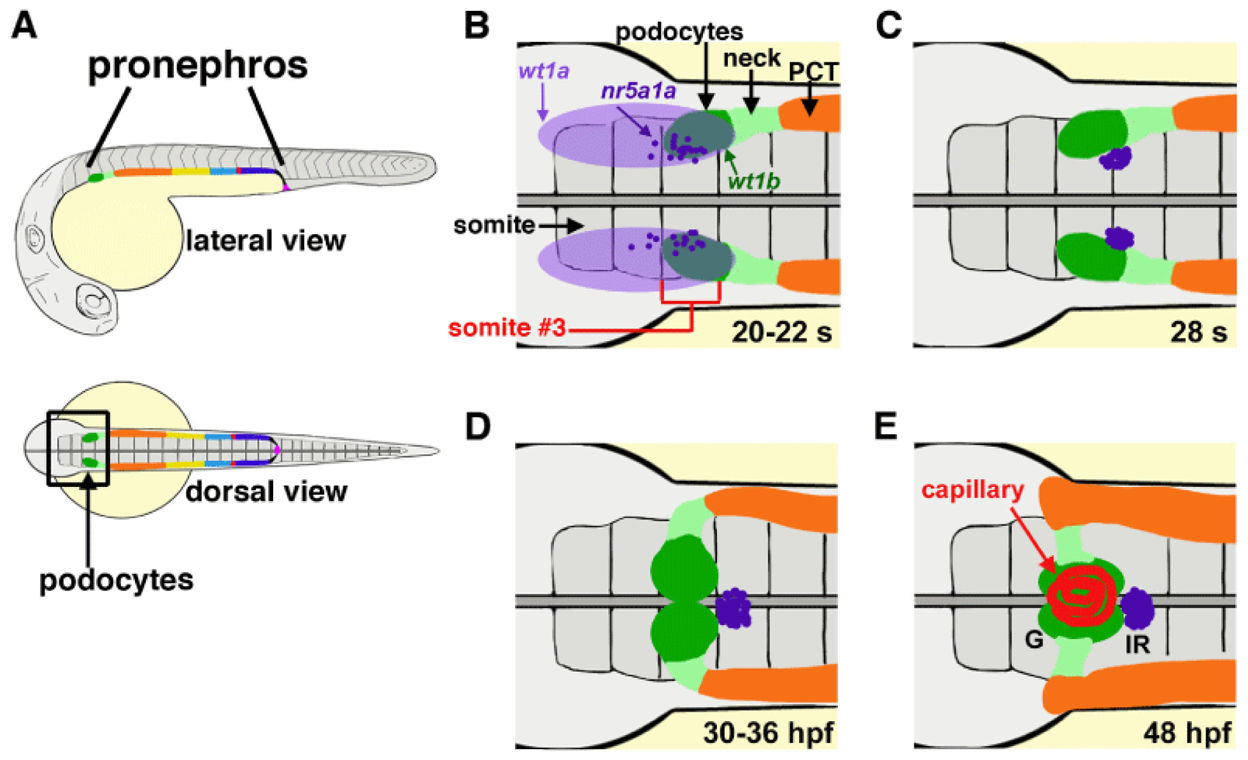
Glomerular development in the zebrafish. (A) The zebrafish pronephros is comprised of a pair of segmented nephrons, and podocytes (dark green) occupy the rostral-most position. (B–E) Anatomy of the cell types that develop in proximity to podocytes over development: (B) 20–22 somite stage; (C) 28 somite stage; (D) 30–36 hours post fertilization; (E) 48 hours post fertilization. Gene expression of *wt1a* (light purple) is broad, while *wt1b* transcripts (dark green) are restricted next to somite (s) three, and interrenal precursors marked by *nr5a1a* transcripts are interspersed in this region. The neck (light green) is located caudal to the podocytes, followed by the proximal convoluted tubule (PCT, orange). Morphogenesis of these populations is progressive from the 20 s stage through to 48 hours post fertilization (hpf), when the podocytes have migrated to the midline and recruited capillaries to form a single glomerulus (G). The interrenal gland (IR) is situated just caudal to the glomerulus. (Images adapted from Ref [16] with author rights).

Due to these fundamental parallels, the zebrafish is a useful model organism to delineate the regulatory networks that are responsible for directing formation of several different nephron cell types [14–22], perhaps first and foremost being podocytes. Zebrafish podocytes exhibit morphological and gene expression characteristics that are very similar with mammalian podocytes [17–21]. The structural simplicity of the zebrafish pronephros provides the benefit of being able to label and visually track podocytes during development (Figure 1) [16]. Furthermore, integrity of the glomerulus can be assessed in the zebrafish embryo, which has facilitated the systematic assessment of factors that are essential to form or maintain the filtration barrier through the utilization of gene knockdown tools (like morpholinos) and gain of function studies (such as cDNA over-expression) [16]. Thus, the continued study of podocyte development using zebrafish has immense potential.

To date, a number of studies have provided insights on the requirements for normal podocyte and/or glomerular development in zebrafish [18–31]. Here, we discuss in depth several contemporary research studies that have used the zebrafish pronephros to elucidate new information about the molecular regulation of podocyte formation during nephrogenesis, with the central focus on a recent study that analyzed the genetic interactions and biochemical activities of the *Wilms’ tumor suppressor-1 (Wt1)* gene during podocyte differentiation.

### Podocyte conservation among vertebrates and the regulation of wt1a/Wt1 homologs by RA signaling

Glomerular podocytes in zebrafish have numerous similarities to mammals, including their ultrastructure, gene expression, and function [17–31]. For example, transmission electron microscopy of zebrafish podocytes has demonstrated that they extend elaborate foot processes and interact with a trilaminar glomerular basement membrane, similar to their mammalian counterparts [17,29]. Further, the gene expression profile of zebrafish podocytes has been shown to mirror that of mammalian podocytes [18,21,23–31].

Among this gene list is *Wt1*, which encodes a mammalian zinc-finger transcription factor and RNA binding protein that is essential for normal renal development and one of the earliest markers of podocytes in vertebrates [7]. Mature and developing podocytes in zebrafish express the *Wt1* paralogs *wt1a* and *wt1b* [18,21,30,31]. In zebrafish, *wt1a* expression is detected first in a broad domain [17,18,26,30], and then transcripts encoding *wt1b* are expressed in a subset of cells within the *wt1a* domain. The dual *wt1a/wt1b*-expressing cells are the podocyte progenitors (Figure 1) [26,30]. Intermingled in the *wt1a*-expressing field are cells that form the interrenal gland, which produces steroid hormones like its mammalian counterpart, the adrenal gland (Figure 1) [28]. Loss of function studies have demonstrated that knockdown of *wt1a* disrupts formation of the glomerulus by leading to a reduction in the number of podocytes that develop [28,31]. The role of *wt1b* has not been fully characterized, and conflicting loss of function studies have been reported to date. Knockdown of *wt1b* has been associated with high incidence of edema (>70%), a phenotype that can indicate renal failure; other researchers have reported that *wt1b* may be dispensable for podocyte development due to redundant roles with *wt1a*, based on knockdown studies in which dual loss of *wt1a/1b* was not more severe than wt1a knockdown alone [21,31].

**Figure 2:**
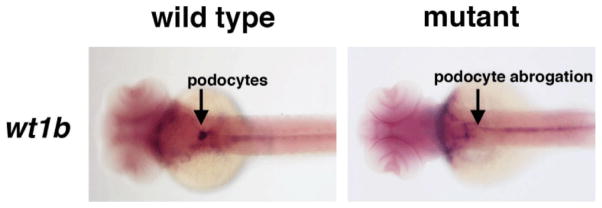
RA deficiency leads to absent or reduced numbers of podocytes in the zebrafish pronephros. Whole mount *in situ* hybridization was performed with a riboprobe to *wt1b* (purple) at the 48 hpf stage of development, when the podocytes have migrated to the midline to form a single glomerulus. Wild type embryos display strong expression in two fused, oval clusters of podocytes at the midline, while *aldh1a2* mutant embryos lack podocytes at this stage.

During zebrafish pronephros formation, the expression of both wt1a and wt1b in renal progenitors is contingent on the presence of Retinoic Acid (RA) signaling [18,19]. RA is a well-established morphogen that elicits dose-dependent effects on target tissues, and RA gradients are essential in many developing tissues [32]. RA leads to changes in gene transcription through binding to heterodimeric complexes of retinoic acid receptors (RARs) and retinoid X receptors (RXRs) [32]. Normal renal progenitor development in the zebrafish pronephros requires RA [18,19]. RA is secreted by paraxial mesodermal cells located adjacent to the intermediate mesodermal field of renal progenitors [18,19]. This source of RA is necessary and sufficient for the patterning of proximal cell fates when the renal progenitors develop, which include both podocytes and the proximal tubule segments [18,19]. Embryos that are deficient in expression of the RA-biosynthesis enzyme *aldehyde dehydrogenase 1a2 (aldh1a2)*, or have been treated with chemical inhibitors to block RA production or downstream signaling, fail to form podocytes or proximal segments [18,19]. For example, side-by-side examination of wt1b expression in *aldh1a2* mutants compared to their wild-type siblings shows that the mutants fail to express *wt1b* in the developing glomerulus (Figure 2). Further, both podocyte and proximal tubule development in *aldh1a2* mutant embryos could be rescued by exogenous treatment with all-trans RA [19]. Interestingly, RA treatment was associated with elevated levels of *wt1a* transcripts in the intermediate mesoderm based on whole mount *in situ* hybridization analysis [18]. However, these studies did not determine whether RA acts directly or indirectly to influence renal progenitors.

A direct link was subsequently established between RA and the promoter of *wt1a/Wt1* [20]. Analysis of the *wt1a* promoter region in zebrafish revealed an upstream enhancer that is highly conserved with the genomic region upstream of *Wt1* in human, mouse, and *Xenopus* [20]. Through biochemical studies, this *wt1a/Wt1* enhancer sequence was shown to be bound by complexes of the RARβ/RXRα, but not by either protein alone [20]. These experiments provide compelling proof of principle evidence that the RAR/RXR proteins can regulate *wt1a/Wt1*, thus suggesting that direct interactions occur between RA and the podocyte gene *wt1a/Wt1*.

## The essential interactions between Wt1, FoxC1/2 transcription factors and Notch signaling components during podocyte development

Recently, insights into the formation of the podocyte lineage, and how *wt1a/Wt1* cooperates with other essential podocyte factors have been elucidated through combinatorial loss of function experiments in zebrafish embryos along with biochemical assays of various zebrafish proteins and their mammalian counterparts [21]. In the subsequent sections, we discuss these studies.

### Spatiotemporal resolution of podocyte precursors within the renal progenitor field

To characterize podocyte development in the zebrafish pronephros, researchers analyzed gene expression from the podocyte progenitor stage until differentiation into a mature podocyte [21]. Whole mount *in situ* hybridization was used to localize the mRNA transcripts of transcription factors characteristic of both early and late stages of podocyte development. The expression patterns of early podocyte markers *wt1a, wt1b, mafba, hey1, lhx1a, pax2a* were determined from 15 somites to 48 hours post fertilization (hpf). Markers that are expressed in mature podocytes, such as *nephrin, podocalyxin, podocin,* and *integrinα3* were also localized at later time points in development (24 hpf, 36 hpf, and 48 hpf). In sum, expression patterns of these markers suggested that podocyte progenitors appear around the 15 somite stage marked specifically by *wt1b* transcripts, and that terminal differentiation proceeds at 24 hpf due to the appearance of transcripts encoding slit diaphragm components like *nephrin* and *podocalyxin*. Additionally, the maturation of podocytes coincides with an upregulation of wt1a and a downregulation of *lhx1a, hey1*, and *pax2a* transcripts.

To gain further insight into the podocyte development process, researchers investigated podocyte formation alongside the neighboring neck, proximal tubule, and interrenal gland tissues [21]. The expression domains of markers characteristic of the three different cell types were analyzed at 24 hpf and 36 hpf. Detection of *wt1b* transcripts was used to label and visualize the podocytes, *pax2a* for the neck region, *cdh17* for the non-podocyte renal epithelia, *slc20a1a* for the proximal convoluted tubule, and *nr5a1a* used to label the interrenal gland cells. The results showed non-overlapping cellular domains, thus establishing the respective spatial domains of each cell group at the rostral end of the pronephros (Figure 1).

Since podocyte progenitors initially express *pax2a*, the origin of podocyte progenitors was determined by a comparison of the expression patterns of *pax2a* and the podocyte marker *wt1a* in a very early stage of development [21]. In the 8 somite stage embryo, the cells expressing both of these markers are the putative podocyte progenitors. Whole mount *in situ* hybridization results showed the most co-expression in the cells of the intermediate mesoderm adjacent to somite 3. By the 15 somite stage, these cells produce a population of podocyte and neck progenitors. In support of these results, laser ablation of one side of the intermediate mesoderm adjacent to somite 3 resulted in reduced *pax2a* expression and close to a complete absence of *wt1b*-expressing podocytes on the ablated side of the embryo at 36 hpf, but normal expression of *nephrin* on the unablated side. This suggests that most of the podocytes originate from the intermediate mesoderm adjacent to somite 3 during zebrafish development (Figure 1).

To further analyze the candidate factors responsible for podocyte specification, expression of several members of the Notch signaling pathway and other transcription factors were analyzed along with podocyte markers [21]. Whole mount *in situ* hybridization of 8 somite stage embryos revealed that the Notch ligands *jag1b* and *jag2a* are expressed in the intermediate mesoderm along with the transcription factor *foxc1a*. The expression domains of these factors overlap with the podocyte markers *wt1a* and *wt1b* in the region of intermediate mesoderm adjacent to somite 3, suggesting that these factors likely have a contribution to podocyte formation.

### Genetic analysis of podocyte patterning

Morpholino knockdowns of *wt1a, foxc1a*, and the Notch mediator *rbpj* were used to delineate their roles in podocyte specification [21]. Under single knockdown conditions it was found that morphant embryos deficient in expression of any one of these three transcripts exhibited a reduced number of *wt1b*-expressing podocyte progenitors at the 15 somite stage and also later at 24 hpf. Although there were *wt1b*-expressing podocytes in the *wt1a, rbpj*, and foxc1a morphants at 24 hpf, they were only abrogated by 36 hpf in the *wt1a* morphants, and did not express normal levels of other markers characteristic of mature podocytes, such as *nephrin* and *podocalyxin*. These results indicate that wt1a plays roles in podocyte maturation and survival. In contrast, *rbpj* and *foxc1a* knockdowns displayed reductions in *nephrin* or *podocalyxin* expression in podocytes at 36 hpf, suggesting that deficiency of these factors alone does not significantly disturb podocyte differentiation. These findings are consistent with amphibian development studies in which the single elimination of *wt1* or *foxc2* was not sufficient to abrogate podocyte development in the pronephros [33]. Interestingly though, dual knockdowns of *wt1* and *foxc2* resulted in the loss of podocytes in amphibians [33].

In keeping with the notion that combinations of these factors may be essential for podocyte specification and/or differentiation, dual knockdowns of *wt1a/rbpj, foxc1a/wt1a*, and *foxc1a/rbpj* in zebrafish embryos all displayed an absence of early and late podocyte marker expression, suggesting that podocyte specification was abrogated [21]. The lack of podocytes was confirmed to be a result of the morpholino knockdown, and not simply due to a loss of the podocyte progenitor cell population of the intermediate mesoderm, because staining for *pax2a* and *myod1* did not show a truncated intermediate mesoderm, and there were no elevated levels of apoptosis in this region.

Further, the effect of various single and double knockdowns on neck and interrenal gland cell fate in the zebrafish pronephros was also evaluated [21]. *rbpj/foxc1a* deficient embryos developed a fairly normal *pax2a*-expressing neck segment, but the *wt1a/rbpj* and *wt1a/foxc1a* morphants exhibited a noticeably reduced *pax2a* expression in the neck segment at 48 hpf. No change in proximal tubule development was observed in the double knockdown embryos. Interestingly, singly deficient *wt1a* and *rbpj* morphants showed an increase in *nr5a1a*-expressing interrenal progenitor cells. In the double *wt1a/rbpj* knockdown, the increase in cell number was even more apparent, suggesting that *wt1a* and *rbpj* may each play a role in the suppression of interrenal gland formation. *foxc1a* morphants did not show a similar enlargement of the interrenal progenitor field, but *foxc1a/rbpj* double deficient embryos exhibited an interrenal gland of modestly increased size. The *foxc1a/wt1a* double knockdowns had an interesting phenotype, in which there was an absence of *nr5a1a*-expressing interrenal gland cells. This suggests that *foxc1a* and *wt1a* may act redundantly to modulate interrenal cell development. The results of the combinatorial knockdowns of *wt1a, rbpj*, and *foxc1a* indicate that the interplay between these three factors governs the development of podocytes, the neck segment, and the interrenal gland.

The expression of a downstream target of the Notch pathway was analyzed under single and combinatorial knock down conditions of the three factors of interest [21]. All double knockdown morphants had no *hey1* expression, and single knockdowns had significantly reduced *hey1* expression. This finding supports a model *whereby wt1a, foxc1a*, and *rbpj* regulate common targets during podocyte development.

## Elucidation of physical interactions between *wt1a/Wt1, foxc1a/FoxC2*, and Notch pathway components in zebrafish and mammals

In order to gain insight into the protein-protein interactions between *wt1a, foxc1a*, and Notch signaling factors, Glutathione S-transferase (GST) tagged *in vitro* pull-down assays were also completed [21]. GST-*rbpj* was able to bind NICD3, the intracellular domain of Notch 3, as well as wt1a, and foxc1a, which was unexpected. Interestingly, protein-protein interactions between GST-*foxc1a* and NICD3 could not be detected. In addition, GST-*wt1a* did not directly interact with NICD3. Despite the lack of interaction with NICD3, both these GST tagged proteins could bind the reciprocal protein partner. These results suggest *rbpj* connects NICD3 with *wt1a* and *foxc1a* using protein-protein interactions during podocyte development, however the data cannot rule out several distinct protein complexes instead of one large multimeric complex. These results were confirmed by co-immunoprecipitation in HEK293T cells, due to the lack of viable zebrafish antibodies. By overexpression of the murine homologs of these proteins, followed by co-immunoprecipitation studies, the results from the pull-down assay were confirmed, suggesting these protein-protein interactions are conserved in mammals.

To investigate the effects of these complexes on promoter activation with the zebrafish genes and their respective mammalian counterparts, cells with the *Hey1* promoter luciferase reporter were co-transfected with *NICD1, foxc1a, FoxC2*, or *Wt1* [21]. Each of these independent experiments showed a 4-fold, or less, induction of luciferase compared to control. Excess *fox1a, Fox2C*, or *Wt1* combined with *NICD1* increased the luciferase activation to between 7 and 11 fold above controls. Similar results were achieved when *fox1a* or *Fox2C* were combined with *Wt1*, without exogenous *NICD1*. The most synergistic result occurred with triple transfections of *NICD1, fox1a/Fox2C*, and *Wt1*, which increased activity of the *Hey1* promoter 13–15 fold. To test this synergy, a synthetic Notch reporter line with several Rbpj binding sites upstream of a minimal promoter was used. NICD1 activated this promoter, while co-transfection of NICD1 and foxc1a or Wt1 inhibited transactivation, thus suggesting these factors antagonize Notch signaling under these conditions. Furthermore, the promoter of a more mature gene expressed in podocytes, Podocalyxin, was tested. NICD1 had very little effect, suggesting the role of Notch occurs early in podocyte development. Conversely, *Wt1* and *Fox*c*1a* induced Podocalyxin promoter activity over controls. Furthermore, the transfection of these factors seems to have dose dependent effects as a 2:1 ratio of *foxc1a* over *Wt1* shows additive effects, however a 5-fold excess of *Foxc1* suppresses activation of the promoter. These results suggest the ratio of these factors is important in the regulation of podocytes during development.

Taken together, these biochemical studies reveal previously unknown physical interactions between *wt1a/Wt1* with *foxc1a/FoxC2, Rbpj*, and *NICD*. Further, these studies suggest that different physical interactions of these proteins are capable of binding genomic targets, and that switches in the complex components over time may orchestrate transcriptional alterations that proceed during podocyte differentiation. These data provide new molecular insights into podocyte development and emphasize the necessity of further biochemical analyses to reveal how transcriptional factors control renal progenitor lineage choices and cell type maturation.

## Conclusion

Progress understanding podocyte development is highly significant to the biomedical community because the loss of podocytes in humans leads to kidney disease. Podocyte structure and molecular composition is conserved between zebrafish and higher vertebrates, such as mice and humans. Therefore, identification of the genetic pathways that are required for podocyte formation in the zebrafish are likely to be broadly applicable to understanding development of this cell type across numerous species. As presented in this commentary, recent cross-species research has provided evidence that RA regulates *wt1a/Wt1* expression, and that *wt1a/Wt1* can interact physically with different co-factors to modulate the transcriptional activity of essential podocyte genes like podocalyxin. Parallels between various genetic and biochemical attributes of Wt homologs across zebrafish, amphibian, and mouse indicates that research with these systems can provide insights into conserved as well as species-specific renal programs of development. Thus, additional podocyte research with the zebrafish model is poised to make useful contributions to this area of nephrology in the years ahead.

Continued work to identify Wt1 targets [34] and to ascertain the full transcriptional profile of podocytes [35,36], is necessary to solve the remaining enigmas of Wt1 function in podocyte ontogeny and identify players in podocyte gene regulatory networks, respectively. Further, ongoing identification of disease-associated loci through genome wide association studies can triage potentially causative genetic components in kidney disease [37]. The zebrafish pronephros model provides a valuable system in which the functional role of such genes can be rapidly assessed with loss and gain of function experiments, as discussed herein. For example, morpholino studies in zebrafish have been implemented to assign critical roles for a number of genes associated with podocyte differentiation or physiological maintenance [38–47]. Insights from such avenues of research can be applied to better understand how podocytes might be generated *in vitro* or *in vivo* for the treatment of chronic kidney disease. The ability to coax human induced pluripotent cells to the podocyte lineage has been reported [48], and further progress on this and similar research endeavors may lead to innovative regenerative therapies for patients with renal conditions.
